# Flexural Strength and Surface Properties of 3D-Printed Denture Base Resins—Effect of Build Angle, Layer Thickness and Aging

**DOI:** 10.3390/ma18040913

**Published:** 2025-02-19

**Authors:** Shaimaa Fouda, Wenjie Ji, Mohammed M. Gad, Maram A. AlGhamdi, Nadja Rohr

**Affiliations:** 1 Department of Substitutive Dental Sciences, College of Dentistry, Imam Abdulrahman Bin Faisal University, Dammam 31441, Saudi Arabia; smfouda@iau.edu.sa (S.F.); mmjad@iau.edu.sa (M.M.G.); maalghamdi@iau.edu.sa (M.A.A.); 2 Biomaterials and Technology, Department Research, University Center for Dental Medicine Basel UZB, University of Basel, Mattenstrasse 40, CH-4058 Basel, Switzerland

**Keywords:** 3D printing, denture bases, flexural strength, mechanical tests, surface properties, complete denture, polymethyl methacrylate

## Abstract

A variety of printable resins for denture bases are available, without detailed instructions on print parameters. This study aimed to evaluate the effect of the printing build angle and the layer thickness of 3D-printed denture base resins before and after thermocyclic aging on flexural strength values and surface properties. The flexural strength, surface roughness (Ra, Rz) and hardness (HM, HV2) of two 3D-printed denture base resins (Formlabs (FL) and V-print dentbase, VOCO, (VC)) were therefore compared to a conventionally pressed cold-curing control material (PalaXpress (PP)). The specimens were printed at a 0°, 45° or 90° build angle and the layer thickness was varied for FL at 50 and 100 µm and evaluated before and after thermocyclic aging (*N* = 200; *n* = 10). Differences in flexural strength values were analyzed using multifactorial ANOVAs (α = 0.05). The build angle and aging significantly affected the flexural strength of the 3D-printed denture base resins (*p* < 0.05), while the layer thickness showed no effect for FL (*p* = 0.461). The required threshold value of 65 MPa defined by ISO 20795-1 was exceeded by PP (70.5 MPa ± 5.5 MPa), by FL when printed at 90° (69.3 MPa ± 7.7 MPa) and by VC at 0° (69.0 MPa ± 4.6 MPa). The choice of an appropriate build angle for each material and printing technology is crucial for the flexural strength and consequently the clinical longevity of a printed denture base.

## 1. Introduction

The use of computer-aided design and computer-aided manufacturing (CAD/CAM) has recently increased for the fabrication of complete dentures. This technology allows denture production in a reduced time, with higher retention and improved mechanical and physical properties compared to conventionally fabricated dentures using the press technique with cold-curing resin [1,2]. The two methods used in implementing CAD/CAM are the subtractive milling of industrially pre-polymerized blanks and additive (3D printing) methods [3]. Pre-polymerized denture base resins for CAD/CAM have superior mechanical properties compared with 3D-printed resin [4]. Nevertheless, restorations fabricated by the additive method are built layer by layer, thus they can reproduce surface details more precisely than the subtractive method. In addition, additive technology can fabricate multiple restorations at the same time, reducing the fabrication time and material waste in comparison to the subtractive method [5,6,7].

Several factors can affect the mechanical and surface properties of additively fabricated dentures, including the printing layer thickness, post-curing time and temperature, printing technology and the addition of reinforcing particles [8,9,10,11,12,13,14,15]. Other factors that can influence the flexural strength of 3D-printed resin are the material viscosity, the thickness of the printing layer and the printing orientation [11]. The effect of the printing build angle on flexural strength may depend on the printer and material, as studies report the highest values at angles of 0° [12], 45° [14] and 90° [11,13]. These results may seem surprising because, from a mechanical perspective, flexural strength values should be lowest at 90° with the cross-linked layers directly exposed to the tension zone, serving as crack initiators.

Also, the wettability of denture base resins is influenced by the printing angle, with more hydrophilic properties reported for printing angles > 70° [16]. There is some controversy in the reporting of the effect of the printing layer thickness. A printing layer thickness of 50 µm resulted in a higher flexural strength and hardness than 100 µm in [11,17,18]. In another study, the highest flexural strength values were measured with a 100 µm printing layer thickness and the highest hardness at 50 μm [19]. In addition, a range of different printable denture base materials is currently available, consisting of different monomers that vary in length and functional groups, affecting numerous factors such as viscosity, polymerization shrinkage, the degree of polymerization or mechanical properties and ultimately long-term stability. Thermocyclic aging between 5° and 55 °C has been commonly employed in dental research since 1952, simulating aging in the oral cavity due to hot or cold food intake [20]. For printed denture base materials, thermocyclic aging has been reported to decrease flexural strength for some printed resin materials, potentially due to water absorption [21]. The variation in the effects of the printing build angle and layer thickness on the flexural strength values of 3D-printed denture base resins does not yet allow one to establish general manufacturing guidelines for clinical applications. Also, the effect of thermocyclic aging on additively manufactured denture base material has not been sufficiently studied.

Therefore, the purpose of this study was to test the effect of the printing build angle and layer thickness of two different 3D-printed denture base resins before and after thermocyclic aging. The outcome will allow dental technicians to choose the optimal build angle and layer thickness when printing denture bases. As a secondary outcome, hardness and surface roughness parameters were obtained. The research hypotheses were that (1) the flexural strength of 3D-printed resins is affected by the printing build angle, (2) the flexural strength of 3D-printed resins is affected by the printing layer thickness and (3) the thermocyclic aging of 3D-printed resin decreases its flexural strength values

## 2. Materials and Methods

Flexural strength specimens of two different denture base resins (Formlabs denture base resin, FormLabs, Somerville, MA, USA (FL) and V-print dentbase, VOCO, Cuxhaven, Germany (VC)) were produced and compared to a conventionally pressed control material (PalaXpress, Kulzer, Hanau, Germany (PP)) (Table 1). Materials were chosen based on compatibility with the available printers, and the control material in the color “clear” has been previously tested, allowing for comparability between studies [22,23].

The printing build angle was 0°, 45° or 90° and layer thickness was varied for the material FL at 50 and 100 µm, as previously chosen by others [11,17,18,19]. Flexural strength was obtained at baseline according to ISO 20795-1 [24] and after thermocyclic aging. Additionally, hardness and surface roughness were measured.

### 2.1. Specimen Preparation

**Figure 1:**
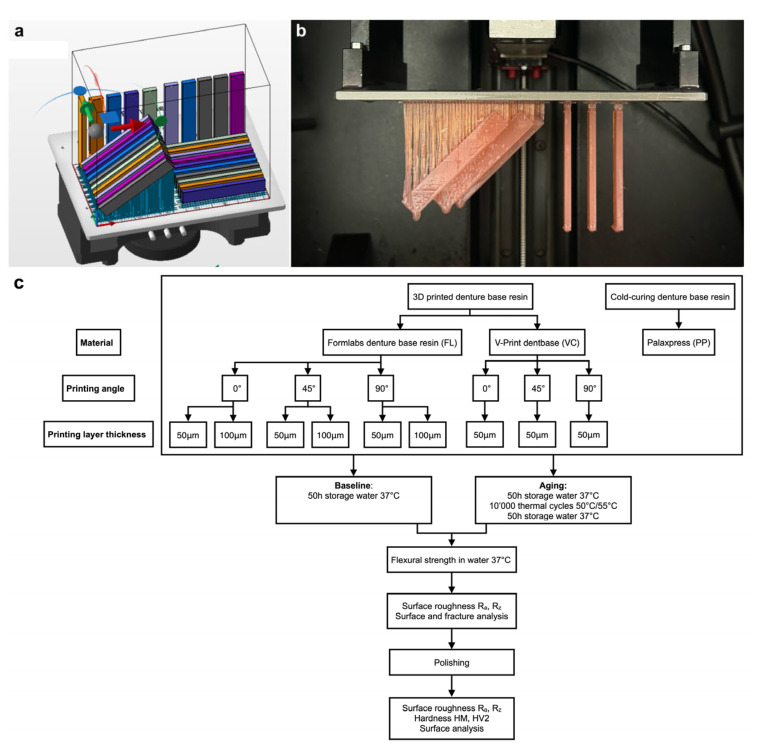
(**a**) Nesting of flexural strength specimens at 0°, 45° and 90° angle, (**b**) printed specimens with support structure in 45° and 90° angulation, (**c**) test set-up overview (*n* = 10 per group).

A bar-shaped specimen with a dimension of 64.0 mm × 10.0 mm × 3.3 mm was virtually designed (123D design, Autodesk, version 2.2.14, San Francisco, CA, USA). A hundred and eighty specimens were nested on the building platform, using support structures in a 0°, 45° or 90° angulation (Figure 1).

Denture base material FL was processed using a stereolithography (SLA) printer (Form 3, FormLabs), while for material VC digital light processing (DLP) print technology (P30 Straumann, Rapid Shape, Heimsheim, Germany) was used. Material FL was additionally printed with layer thicknesses of 50 or 100 µm for each printing orientation. For VC, it was only possible to print the material at 50 µm due to the limited printer settings. After printing, the specimens were cleaned 3 times in 99% isopropyl in an ultrasonic bath (TPC-15; Telsonic, Bronschhofen, Switzerland) for 5 min. Support structures were removed manually, and post-polymerization was conducted for 2 × 2000 flashes (Otoflash G171, NK-Optik, Baierbrunn, Germany).

A conventionally pressable resin material PP served as the control. Specimens were produced using a silicon mold. Manufacturer’s instructions were followed for the monomer–polymer ratio, mixing and polymerizing methods. A pressure vessel was utilized for polymerization in water at 55 °C and at 1 bar of pressure for 30 min. After withdrawing the PP specimens from the mold, excess material was carefully removed using silicon carbide paper grit 1200 (Struers, Ballerup, Denmark).

A total of 200 specimens were produced, and all were stored for 50 h at 37 °C. To simulate aging, half of the specimens of each group were subsequently subjected to 20,000 thermal cycles between 5 °C and 55 °C, with a dwell time of 30 s (THE-1100, Mechatronik, Feldkirch, Germany). After completing the thermal cycling, the specimens were stored again for 50 h in distilled water at 37 °C.

### 2.2. Flexural Strength

The flexural strength was obtained using a universal testing machine (Z020, Zwick/Roell, Ulm, Germany). The specimens, having been immersed in a water bath at 37 °C for 50 h prior to testing, were subsequently gauged and then placed in a water bath of 37°C in a custom-made device during loading (*n* = 10 per group) following ISO 20795-1 [24]. The specimens underwent vertical load application at their center until fracture with a crosshead speed of 5 mm/min, and their flexural strength was determined using the following formula:

Flexural strength = 3FL/2bh2

F = maximum force in newtons (N); L = distance between supports, which was 50 mm; b = width of the tested specimen; and h = specimens’ thickness.

### 2.3. Surface Analysis

The failure sites were visualized after flexural strength testing using light microscopy (Wild M7A, Wild Heerbrugg, Gais, Switzerland). The surface morphology of one specimen per group was additionally recorded using scanning electron microscopy. Specimens were mounted and then sputtered with gold at 20 mA for 440 s (Balzers Sputter Coater SCD 050, BAL-TEC, Balzers, Liechtenstein). The morphology was inspected under a scanning electron microscope (Philips XL30 ESEM, Eindhoven, The Netherlands) at ×500 magnification at 5 kV.

### 2.4. Surface Roughness

The surface roughness parameters R_a_ (arithmetical mean height) and R_z_ (maximum height) were measured per specimen using an optical confocal profilometer (VK-X1050 Keyence, Osaka, Japan) with 10 parallel lines over 500 µm (20× objective, field of view 709 µm × 532 µm). To evaluate polishability, the same pieces were treated using silicon carbide paper P1200, P2500 and P4000 with permanent water irrigation using a polishing machine (Minitech 265, Presi, Hagen, Germany) for 20 s each. Surface roughness parameters were reassessed after polishing.

### 2.5. Hardness

Martens (HM) and Vickers hardness (HV2) were measured using a Vickers tester (Z2.5, Zwick/Roell, Ulm, Germany). Each polished piece, as previously used for surface roughness measurements, was subjected to a load of 19.61 N for 5 N/s at 3 different sites; the force was held for 2 s (*n* = 30 measurements per group). The HV2 was determined by adjusting the impression boarders of the indentation using microscopic imaging.

### 2.6. Statistical Analysis

The number of specimens was increased to 10 per group of the 5 specimens recommended by ISO 20795-1 [24] to additionally consider aging effects. Data are reported descriptively with mean and standard deviations. The data were tested for normal distribution using the Shapiro–Wilk test (α = 0.05). Multifactorial ANOVAs were applied as follows for flexural strength values: A three-way ANOVA to all measurements of group FL to test for the effects of build angle, layer thickness and aging. A three-way ANOVA to group FL, VC at 50 µm to test for effects of build angle, material and aging. A two-way ANOVA to group FL, VC at 50 µm, 45° and PP to test for the effects of material and aging (StatPlus:mac Pro v6.1.25, AnalystSoft, Alexandria, VA, USA).

## 3. Results

### 3.1. Flexural Strength

**Figure 2:**
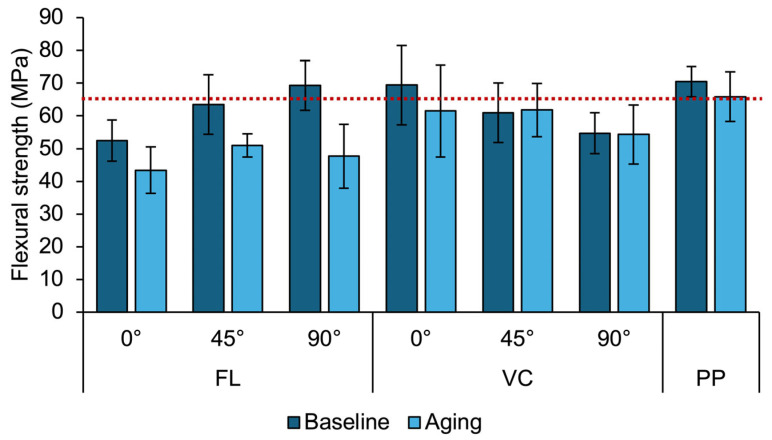
Flexural strength mean and standard deviations of denture base resin materials FL and VC at build angles of 0°, 45° or 90° and conventionally pressed material PP. Printing layers of 50 µm and 100 µm for FL were pooled, as there were no significant differences (*p* < 0.001). The red line indicates the required flexural strength of 65 MPa based on ISO 20795-1.

Flexural strength values within groups were normally distributed; therefore, multifactorial ANOVAs were chosen for the statistical analysis. The flexural strength values of the respective groups and the ANOVA statistics are given in Table 2 and Table 3. The three-way ANOVA applied to group FL revealed a significant effect for the build angle (*p* < 0.001) and aging (*p* < 0.001) but not for layer thickness (*p* = 0.461). Therefore, for the graphical visualization in Figure 2, data for 50 µm and 100 µm printing layer thicknesses were pooled. Significantly higher flexural strength values for FL were achieved overall (before and after aging) when the specimens were printed at a 45° (58.0 MPa ± 11.5 MPa) or 90° (58.5 MPa ± 13.9 MPa) angle (*p* = 0.972) than for the 0° (48.0 MPa ± 9.1 MPa) angle (both *p* < 0.001). Thermocyclic aging significantly decreased the flexural strength of FL from 61.8 MPa ± 11.0 MPa at baseline to 47.9 MPa ± 10.0 MPa after aging.

For the three-way ANOVA comparing the printed materials FL and VC at a 50 µm layer thickness, a significant effect was again observed between materials (*p* = 0.003) for the build angle (*p* = 0.014) and aging (*p* < 0.001). Flexural strength values were overall significantly higher for material VC (60.4 MPa ± 10.6 MPa) than FL (55.4 MPa ± 13.2 MPa) when build angles and aging were pooled. For the build angle, the highest values overall for both materials when pooled at 45° (60.7 MPa ± 9.4 MPa) and 90° (58.2 MPa ± 12.8 MPa) (*p* = 0.445) were found. Values at 0° (54.8 MPa ± 13.5 MPa) were significantly lower than at 45° (*p* = 0.014) but not than at 90° (*p* = 0.241).

To allow for a material comparison with material PP, flexural strength values at a 45° angle of FL and VC were selected, as those angles displayed the second highest flexural strength values and most consistent values after aging. A two-way ANOVA was conducted comparing materials FL and VC printed at a 50 µm layer thickness at an angle of 45° with the conventionally pressed material PP, revealing a significant difference between materials (*p* = 0.007) but not for aging (*p* = 0.118). Significantly higher values were measured for PP (68.2 MPa ± 6.1 MPa) than VC (61.4 MPa ± 9.9 MPa) (*p* = 0.046) or FL (60.0 MPa ± 9.1 MPa) (*p* = 0.013), which did not differ significantly from each other when printed at 45° (*p* = 0.874).

### 3.2. Surface Analysis

**Figure 3:**
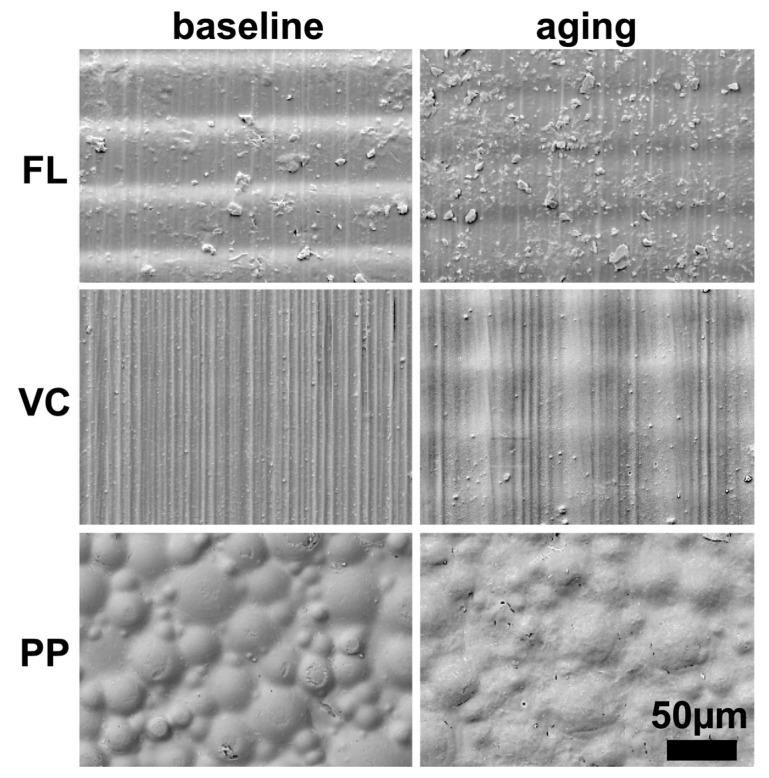
Scanning electron microscope images of specimen surfaces of printed resins FL and VC at 0° angle and 50 µm layer thickness and conventionally pressed material PP before and after thermocyclic aging.

SEM images of the specimen surfaces of printed resins FL and VC at a 0° angle and 50 µm layer thickness and conventionally pressed material PP before and after aging are given in Figure 3. Those images were chosen as, overall, characteristic differences were observable among the materials and due to aging. For the different printing thicknesses, of course the distance between layers changed, and the build angle affected the layer orientation. On images of FL and VC, a wavy structure is observable due to the printing procedure. Particles are present on the surfaces of FL. With PP, round fillers with a size of 5 to 50 µm are visible. Deterioration of the surfaces is observable after aging, indicating swelling.

**Figure 4:**
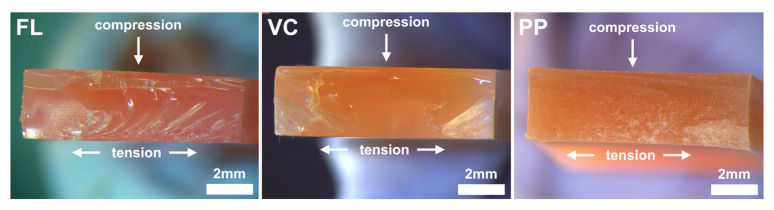
Light microscope images of the fracture sites of the printed resin materials FL and VC and conventionally pressed material PP. Specimens displayed typical compression curls below the arrow of the compression zone.

Light microscope images of fracture sites of the printed resin materials FL and VC and conventionally pressed material PP are displayed in Figure 4. Fractures occurred after deformation of the specimens and initiated from the tension zone. Fracture lines are clearly visible for FL and VC as an indicator of the intrinsic accumulation of stress that resulted in a brittle fracture. For PP, fracture line progression is less pronounced, indicating a ductile fracture mode. All specimens displayed a typical compression curl below the arrow of the compression zone, marking the end of the fracture occurrence.

### 3.3. Surface Roughness

The surface roughness values for parameters Ra and Rz before and after polishing are displayed in Table 4. Initial surface roughness parameters varied among different groups, with an overall R_a_ mean of 2.44 µm ± 1.05 µm and R_z_ mean of 21.30 µm ± 6.57 µm. Maximum R_a_ values were found for group FL at a 0°, 100 µm baseline (4.44 µm ± 1.08 µm) and lowest for group FL at 90°, 50 µm, aging (1.39 µm ± 0.40 µm).

After polishing, a mean R_a_ value of 0.23 µm ± 0.03 µm and an R_z_ of 2.93 µm ± 0.28 µm was measured for printed materials FL and VC, while the surface roughness parameters of the conventionally pressed material PP were slightly lower (R_a_ = 0.15 µm ± 0.02 µm, R_z_ = 1.94 µm ± 0.01 µm). R_a_ and R_z_ correlated linearly before (y = 5.807x + 7.628; R^2^ = 0.808) and after polishing (y = 8.775x + 0.887; R^2^ = 0.830).

### 3.4. Hardness

HM and HV2 values are presented in Table 2. The overall mean and standard deviation were calculated, which were an HM of 131 N/mm^2^ ± 10 N/mm^2^ and HV2 of 22.7 ± 1.1. The linear correlation trend between flexural strength values and HM was weak (y = 0.296x + 114.050; R^2^ = 0.085).

## 4. Discussion

A variety of printable resins for denture bases are available, though detailed instructions on print parameters are often lacking. The inconsistent effects of printing build angle and layer thickness on the flexural strength did not yet allow for the creation of general manufacturing guidelines for clinical applications. The present study therefore tested the effects of build angle, layer thickness and artificial aging on the flexural strength and surface properties of denture base resins fabricated by SLA (FL) and DLP (VC) printing technologies. The first study hypothesis was accepted, because the printing build angle affected the flexural strength of the 3D-printed resins. The second hypothesis was rejected as the printing layer thickness did not affect flexural strength values. The third hypothesis was accepted because thermocyclic aging decreased the flexural strength values of the 3D-printed resins.

The build angle affected the flexural strength of the tested materials, with the highest values for specimens of FL printed at a 90° angle and for VC at a 0° angle. Previous studies confirmed the significant effect of the build angle on flexural strength values [11,12,13,14]. From a mechanical point of view, it might be expected that specimens printed at a 90° angulation would have the lowest flexural strength, as the fusion area of the layers is within the loading zone. This was confirmed with VC, which was printed using DLP technology, where each layer is cured simultaneously by light projection. The highest values of FL at a 90° baseline indicate a strong bond between the printed layers formed due to the repetitive light beam applied to a small area per printing layer with SLA technology. For FL, the 0° values may have been lower due to the more rapid printing and less light exposure per specimen. These results emphasize the importance of selecting the appropriate build angle for each material and printing technology.

The effect of layer thickness was not significant for the flexural strength values of the FL specimens, confirming previous results where an interim resin was tested [25]. Unfortunately, material VC could not be printed with different layer thicknesses due to standardized printer settings. As material PP was conventionally processed, the test set-up was not uniform, this being considered a limitation of this study. Higher flexural strength values and hardnesses of 3D-printed materials at 25 μm or 50 μm than at a 100 μm layer thickness have been reported [11,17,18,26]. Alshamrani et al. found a higher flexural strength at a 100 μm and a higher hardness at a 50 μm printing layer thickness of 3D-printed resin [19]. In the present study, hardness parameters HM and HV2 were within the same range for all groups. HV2 measurements consider the plastic deformation only, while for HM measurements elastic and plastic work are incorporated. As the surfaces were polished to be able to assess the hardness properly, surface defects due to aging may have been removed. The polishing of resins also results in a smearing effect of the surface that may have masked the effect of the printing layers. Therefore, also, no correlation between flexural strength values and HM was found.

The reduction in flexural strength values due to thermal aging confirms the findings of previous studies [9,26,27]. The change in temperatures resulted in the expansion and compression of the specimens. The exposure to water led to water diffusion between the printing layers, thus decreasing flexural strength values [27]. Swelling of the surfaces was also observable in SEM images. Unfortunately, water sorption was not assessed, which would have provided additional information to interpret the study findings [28]. The effect of thermocyclic aging was more severe for FL specimens, while flexural strength values remained stable for VC printed at 45° and 90°. Nevertheless, for both materials the flexural strength values following aging were below the proposed threshold value of 65 MPa indicated by ISO 20795-1 [24]. Flexural strength values at baseline of FL printed at 90° and VC at 0° superseded 65 MPa. The conventionally pressed material PP fulfilled the requirements before and after aging. The fracture mode of the PP specimens was ductile, potentially allowing them to absorb more stress before fracture compared to the printed specimens, where brittle fractures were observed. The applied number of thermal cycles (20,000) corresponded to two years of simulated clinical use [26,29]. ISO 20795-1 recommends flexural strength testing in a water bath of 37 °C, as was done in this study [24]. Geiger et al. found that the flexural strength of resin specimens tested in water was lower than those tested in air at 23 °C [21]. This emphasizes the importance of providing measuring conditions simulating the clinical environment to estimate the mechanical performance of denture base materials when in use.

A low surface roughness below Ra < 0.2 µm as defined by Bollen et al. is crucial to prevent plaque accumulation in the oral cavity [30]. Especially for denture base materials, where the surface is in direct contact with the mucosa, a low surface roughness is crucial, also to prevent candida formation [31]. While the initial surface roughness on all surfaces displayed an overall R_a_ mean of 2.44 µm ± 1.05 µm, polishing decreased the mean R_a_ value to 0.23 µm ± 0.03 µm for the printed and to R_a_ = 0.15 µm ± 0.02 µm for the conventionally pressed surface. However, when producing dentures, this area is generally not polished to not impede the fit of the denture. Unpolished printed resin surfaces may also contain a higher degree of unlinked monomers, having cytotoxic effects [22]. In a previous study, an industrially pre-polymerized resin for the CAD/CAM milling of dentures revealed a higher cell viability of keratinocytes compared with a conventionally pressed cold-curing material [32]. In addition, printable 3D resins are based on methyl methacrylate, which if not sufficiently polymerized can trigger hypersensitivity, asthmatic reactions, local neurological symptoms, irritant and local dermatological reactions [33]. Hence, further improvements on the material properties of 3D-printed resins are necessary, especially regarding post-treatments, providing a smooth surface with a high degree of polymerization to prevent health risks for the patients [34]. The following points are considered limitations and should be improved for future set-ups: Printing at a 100 µm layer thickness was not possible for both materials due to the closed printer settings. Also, water sorption was not evaluated and may have provided further insights into aging behavior.

Nevertheless, the results of this laboratory study revealed that the effect of build angle on flexural strength and surface properties varies among 3D-printed denture base resin materials. Therefore, dental technicians should be aware of the optimum printing angle for each material to obtain the highest flexural strength of the final restoration. As the obtained properties were measured using standardized geometrical specimens, further simulations e.g., using finite element analysis with denture shapes, are needed to draw further conclusions for setting optimal printing parameters.

## 5. Conclusions

Within the limitations of the present study, the following could be concluded:

- The build angle affected the flexural strength of 3D-printed denture base resins, with highest values for material FL printed at a 90° angle and for VC at a 0° angle fulfilling the requirements of ISO 20795-1;
- The printing layer thickness showed no effect on flexural strength values for material FL;
- Thermocyclic aging reduced flexural strength values for printed materials FL and VC, while no significant effect was determined for the conventionally pressed control material PP;
- The conventionally pressed control material PP achieved the highest flexural strength values overall. The flexural strength values of the printed resin denture base materials FL and VC were affected by the printing angle and aging.

## Figures and Tables

**Table 1:** Materials used for flexural strength specimens.

Type	Code	Name	Manufacturer	LOT-Nr.	Composition
Printable denture base resin	FL	Formlabs Denture Base Resin	FormLabs, Somerville, MA, USA	BF20C30O	55–75 weight% urethane dimethacrylate, 15–25 weight% methacrylate monomers, <0.9 weight% phenyl bis (2,4,6-trimethylbenzoyl)-phosphine oxide
Printable denture base resin	VC	V-Print dentbase	VOCO, Cuxhaven, Germany	2402523	50–100% aliphatic urethane dimethacrylate, 25–50% 2,2-bis(4-(2-methacryl-oxyethoxy)phenyl)propane, 5–10% triethylenglycoldimethacrylat, diphenyl(2,4,6), 1-2,5% trimethylbenzoyl)phosphinoxid
Cold-curing denture base resin	PP	PalaXpress	Kulzer, Hanau, Germany	M010244	methyl methacrylate, dimethacrylate, methyl methacrylate-copolymer

**Table 2:** Flexural strength, Martens hardness (HM) and Vickers hardness (HV2) mean and standard deviations of printed materials FL and VC and conventionally pressed denture base resin material PP.

Material	Build Angle	Layer Thickness	Aging	Flexural Strength (MPa)	HM (N/mm^2^)	HV2
FL	0°	50 µm	Baseline	51.0 ± 6.3	136 ± 16	24.5 ± 1.1
			Aging	37.6 ± 7.1	123 ± 15	23.5 ± 1.7
		100 µm	Baseline	54.0 ± 9.1	131 ± 24	23.9 ± 1.8
			Aging	49.3 ± 3.6	140 ± 10	24.6 ± 2.3
	45°	50 µm	Baseline	63.4 ± 7.6	113 ± 34	23.3 ± 1.3
			Aging	56.7 ± 9.7	118 ± 20	22.0 ± 1.6
		100 µm	Baseline	63.6 ± 12.2	126 ± 17	21.6 ± 1.8
			Aging	48.3 ± 9.9	135 ± 13	22.4 ± 1.7
	90°	50 µm	Baseline	71.7 ± 9.1	139 ± 19	21.9 ± 1.4
			Aging	51.9 ± 8.1	130 ± 18	22.0 ± 1.6
		100 µm	Baseline	66.9 ± 6.3	121 ± 25	21.1 ± 2.0
			Aging	43.4 ± 9.0	115 ± 26	22.1 ± 1.4
VC	0°	50 µm	Baseline	69.0 ± 4.6	138 ± 11	21.7 ± 2.5
			Aging	61.6 ± 7.6	141 ± 15	22.9 ± 1.8
	45°	50 µm	Baseline	61.0 ± 5.6	132 ± 14	21.1 ± 1.9
			Aging	61.8 ± 13.3	126 ± 21	22.1 ± 1.4
	90°	50 µm	Baseline	54.7 ± 6.7	133 ± 21	21.9 ± 3.7
			Aging	54.4 ± 15.3	135 ± 22	22.9 ± 1.8
PP			Baseline	70.5 ± 5.5	141 ± 15	23.2 ± 0.8
			Aging	65.9 ± 6.6	150 ± 9	23.1 ± 0.8

**Table 3:** ANOVA tables for flexural strength value comparison.

Factor	DF	SS	MS	F	*p*-Level
Three-way ANOVA (all FL)					
Build angle	2	2821	1410	19.87	<0.001
Layer thickness	1	39	39	0.55	0.461
Aging	1	5788	5788	81.55	<0.001
Build angle × Layer thickness	2	1106	553	7.79	0.001
Build angle × Aging	2	918	459	6.46	0.002
Layer thickness × Aging	1	10	10	0.14	0.706
Build angle × Layer thickness × Aging	2	403	201	2.84	0.063
Within groups	108	7666	71		
Total	119	18,750	158		
Three-way ANOVA (FL 50 µm, VC 50 µm)					
Material	1	765	765	9.68	0.002
Build angle	2	711	355	4.45	0.014
Aging	1	1831	1831	22.95	<0.001
Material × Build angle	2	4179	2090	26.18	<0.001
Material × Aging	1	906	906	11.35	0.001
Build angle × Aging	2	361	180	2.26	0.109
Material × Build angle × Aging	2	275	137	21.72	0.184
Within groups	108	8620	80		
Total	119	17,648	148		
Two-way ANOVA (FL 45° 50 µm, VC 45° 50 µm, PP)					
Material	2	766	383	5.39	0.007
Aging	1	180	180	2.53	0.118
Material × Aging	2	150	75	1.05	0.356
Within groups	54	3839	71		
Total	59	4934	84		

**Table 4:** Surface roughness parameters Ra and Rz before and after polishing (mean and standard deviation) of printed materials FL and VC and conventionally pressed material PP.

Material	Build Angle	Layer Thickness	Aging	Before Polishing R_a_ (µm)	Before Polishing R_z_ (µm)	After Polishing R_a_ (µm)	After Polishing R_z_ (µm)
FL	0°	50 µm	Baseline	1.51 ± 0.30	17.81 ± 2.55	0.25 ± 0.03	3.14 ± 0.36
			Aging	1.89 ± 1.39	19.63 ± 8.64	0.22 ± 0.03	2.80 ± 0.49
		100 µm	Baseline	4.44 ± 1.48	32.36 ± 8.00	0.26 ± 0.05	2.97 ± 0.50
			Aging	4.37 ± 1.69	29.52 ± 8.39	0.26 ± 0.03	3.19 ± 0.33
	45°	50 µm	Baseline	2.02 ± 1.41	23.13 ± 8.83	0.21 ± 0.04	2.68 ± 0.50
			Aging	2.07 ± 2.86	22.73 ± 19.75	0.21 ± 0.03	2.77 ± 0.34
		100 µm	Baseline	4.02 ± 1.54	30.64 ± 9.35	0.22 ± 0.03	2.68 ± 0.35
			Aging	4.43 ± 0.96	34.46 ± 9.61	0.34 ± 0.05	3.72 ± 0.50
	90°	50 µm	Baseline	1.46 ± 0.77	18.54 ± 8.00	0.22 ± 0.03	2.81 ± 0.60
			Aging	1.39 ± 0.40	16.52 ± 3.89	0.23 ± 0.03	3.05 ± 0.32
		100 µm	Baseline	2.63 ± 1.00	24.31 ± 5.21	0.23 ± 0.04	2.80 ± 0.38
			Aging	2.78 ± 0.75	22.98 ± 5.01	0.22 ± 0.04	2.83 ± 1.95
VC	0°	50 µm	Baseline	1.70 ± 0.92	13.95 ± 7.13	0.24 ± 0.04	3.39 ± 0.61
			Aging	1.39 ± 0.39	10.62 ± 3.03	0.18 ± 0.02	2.56 ± 0.45
	45°	50 µm	Baseline	2.55 ± 0.95	21.12 ± 5.10	0.23 ± 0.05	2.83 ± 0.43
			Aging	1.62 ± 0.74	14.00 ± 6.99	0.20 ± 0.01	2.93 ± 0.76
	90°	50 µm	Baseline	1.82 ± 0.55	15.96 ± 4.23	0.20 ± 0.02	2.80 ± 0.54
			Aging	1.81 ± 0.69	16.61 ± 6.72	0.22 ± 0.02	2.78 ± 0.25
PP			Baseline	2.70 ± 0.83	31.13 ± 13.98	0.13 ± 0.01	1.94 ± 0.37
			Aging	2.13 ± 0.61	19.94 ± 5.37	0.16 ± 0.03	1.95 ± 0.36

## Data Availability

The original contributions presented in this study are included in the article. Further inquiries can be directed to the corresponding author.

## Abbreviations

The following abbreviations are used in this manuscript:

FL	Formlabs Denture Base Resin, 3D-printable denture base resin
VC	V-Print dentbase, 3D-printable denture base resin
PP	PalaXpress, conventionally pressed denture base resin
